# The social predictors of paternal antenatal mental health and their associations with maternal mental health in the Queensland Family Cohort prospective study

**DOI:** 10.1007/s00737-022-01257-1

**Published:** 2022-08-19

**Authors:** Barnaby J. W. Dixson, Danielle Borg, Kym M. Rae, Koa Whittingha, Brenda Gannon, Steven M. McPhail, Hannah E. Carter, Karen M. Moritz, Roslyn N. Boyd, Samudragupta Bora, Sailesh Kumar, Julanne Frater, Daniel Schweitzer, Paul Miller, Divya Mehter, Vicki L. Clifton

**Affiliations:** 1 School of Health and Behavioural Sciences, University of the Sunshine Coast, 90 Sippy Downs Drive, Sippy Downs, QLD 4556 Australia; 2 School of Psychology, The University of Queensland, Brisbane, QLD Australia; 3 Mater Research Institute, Aubigny Place, South Brisbane, QLD Australia; 4 Faculty of Medicine, The University of Queensland, Herston, QLD Australia; 5 Queensland Cerebral Palsy and Rehabilitation Research Centre, Child Health Research Centre, The University of Queensland, Brisbane, QLD Australia; 6 School of Economics and Centre for the Business and Economics of Health, University of Queensland, Brisbane, QLD Australia; 7 Australian Centre for Health Services Innovation and Centre for Healthcare Transformation, School of Public Health and Social Work, Queensland University of Technology, Brisbane, Australia; 8 Clinical Informatics Directorate, Metro South Health, Brisbane, Australia; 9 School of Biomedical Sciences, The University of Queensland, Brisbane, Australia; 10 Mothers, Babies and Women’s Health Program, Faculty of Medicine, Mater Research Institute, The University of Queensland, South Brisbane, QLD Australia; 11 Emotional Health Unit, Mater Misericordiae Health Services Brisbane Ltd, Brisbane, QLD Australia; 12 Mater Centre for Neurosciences, Brisbane, Australia; 13 Faculty of Health, School of Biomedical Sciences, Centre for Genomics and Personalised Health, Queensland University of Technology, Kelvin Grove, Brisbane, QLD Australia; 14 Translational Research Institute, Brisbane, QLD Australia

**Keywords:** Parenthood, Mental health, Fathers, Mothers, Social support

## Abstract

Antenatal depression (AND) affects 1 in 10 fathers, potentially negatively impacting maternal mental health and well-being during and after the transition to parenthood. However, few studies have assessed the social predictors of paternal AND or their possible associations with maternal mental health. We analysed data from 180 couples participating in the Queensland Family Cohort longitudinal study. Both parents completed surveys measuring mental health, relationship quality, social support, and sleep quality at 24 weeks of pregnancy. Mothers also completed the same surveys 6 weeks’ postpartum. Antenatal depression, stress, and anxiety were highest among fathers reporting lower social support and higher sleep impairment. Maternal AND, stress, and anxiety were higher among mothers reporting higher physical pain and poor sleep quality. Postnatally, mothers reporting lower social support also reported higher depression, anxiety, stress, and psycho-social well-being. While there were no significant associations between AND among fathers and maternal antenatal or postnatal depression, an exploratory analysis revealed that mothers whose partners reported lower antenatal social support also reported lower postnatal social support and higher postnatal depression. Our findings highlight the importance of including data among fathers to achieve a whole family approach to well-being during the transition to parenthood.

**Supplementary Information:**

The online version contains supplementary material available at 10.1007/s00737-022-01257-1.

Fatherhood has changed dramatically over the last 50 years; men are becoming increasingly involved during their partner’s pregnancy, attending births, and caring for infants (Bakermans-Kranenburg et al. 2019). The support provided by fathers improves maternal emotional stability, mental well-being, and physical health (Mezulis et al. 2004). The contribution of fathers to child rearing positively influences their children’s social, behavioural, and cognitive development including linguistic capabilities, academic performance, and emotional stability (Lucassen et al. 2011). In turn, fatherhood may positively influence some men’s long-term health and social stability in the family (Bakermans-Kranenburg et al. 2019).

Although fathers positively influence family well-being, increased paternal investment coincides with mounting evidence that fathers experience antenatal depression (AND) and postnatal depression (PND) at rates ranging from 1 to 10% globally (Cameron et al. 2016; Paulson and Bazemore 2010; Rao et al. 2020). The wide range of AND and PND incidence reported in the literature may be attributable to both genuine differences between cultures and societies (Cameron et al. 2016; Paulson and Bazemore 2010; Rao et al. 2020) as well as methodology-related factors, with a recent meta-regression indicating that higher study quality was associated with higher incidence estimates (Rao et al. 2020). The trajectory of paternal AND and PND during pregnancy leading into the first 1000 days of parenthood is nonlinear, being high in the first trimester, dropping in the third trimester, and becoming highest at 6–8 months postpartum (Kingston et al. 2018). Notably, some of the peaks in men’s depression coincide with the periods in their children’s lives when maternal attachment and bonding is established (Bakermans-Kranenburg et al. 2019). Infants born to fathers suffering from PND are at higher risk of developing greater emotional hostility, hyperactivity, and anxiety at 3–5 years (Ramchandani et al. 2005, 2011) and are more likely to suffer from depression during childhood (Gentile and Fusco 2017) and young adulthood (Gutierrez-Galve et al. 2019).

In addition to influencing infant, toddler, and child development, paternal mental health impacts maternal mental health and well-being during this period. Paternal PND is positively associated with PND in mothers (Paulson et al. 2016), with meta-analyses showing medium effect sizes (*r* = 0.31) (Paulson and Bazemore 2010). While many fathers in high-income countries are increasingly involved in childcare, the amount of direct paternal infant care varies, and this contributes to maternal mental health. For example, postnatal stress among Korean mothers was lower when perceived and actual paternal investment was greater (Kim et al. 2016). Direct paternal infant care in the first month postpartum resulted in a four-fold reduction in the risk of depression among Taiwanese stay-at-home mothers, resulting in long-term beneficial effects on maternal well-being (Lin et al. 2017). In contrast, unemployed mothers are 3 times more likely than mothers with employment to experience postnatal stress if they have low paternal care (Lin et al. 2017). During the antenatal stage, men may manifest clinical depression, setting the tone for paternal and maternal mental health trajectories during early parenthood.

Maternal AND and PND are well-documented and their underlying biological, social, and economic correlates well-established. These include age, genetic predispositions, adverse childhood conditions, poor relationship quality, low social support from social networks, and lower membership of social groups within their community (Beck 1996; 2001; Manuel et al. 2012). Thus, maternal AND and PND are higher among younger women aged 24 years or younger, decreasing as maternal age increases leading into a marked decline in mental health among mothers over 35 years of age (Guintivano et al. 2018). Such effects may also be more pronounced among first-time mothers and mothers with twins (Bradshaw et al. 2022). Age-related changes in maternal mental health may, in some cases, be attributed to changes in women’s social support, with low social support predicting the likelihood of pre-term births (McDonald et al. 2014). Maternal PND shows 50% heritability, demonstrating that half the variation in PND is genetic (Viktorin et al. 2016). Most recently, genome-wide association research reveals that maternal AND and PND is higher among women with higher polygenic risk of other major depressive disorders (Kiewa et al. 2022).

Despite increasing awareness that declines in paternal mental health during the transition to fatherhood impact maternal and infant mental health, few studies have identified the predictors of paternal AND (Bakermans-Kranenburg et al. 2019; Glasser and Lerner-Geva 2019). Among 157 first-time couples, perinatal mood and relationship satisfaction were the strongest predictors of postnatal depression among fathers (Matthey et al. 2000). Perinatal depression was higher among Finnish men with lower education (Korja et al. 2018), among New Zealand men with high self-reported stress and lower physical health (Underwood et al. 2017), and higher in Polish men with high anxiety (Kiepura and Kmita 2020). Sleep patterns are also strong predictors of paternal declines in mental health, whereby men who sleep 5–6 h per night are at highest risk of AND and PND (Krueger and Friedman 2009; Wynter et al. 2020). Finally, PND was higher among Chinese men reporting lower social support and poor relationship quality (Gao et al. 2009; Mao et al. 2011). Taken together, these studies suggest that relationship quality, social support, and sleep patterns are negatively associated with men’s mental health during the transition to parenthood (O’Brien et al. 2017).

The growing recognition of paternal AND calls for further research into the risk factors associated with declines in mental health among fathers. Highlighting these risk factors is crucial for early identification and prevention, benefiting not only fathers but also improving their relationships with mothers and the developmental trajectories of their children (O’Brien et al. 2017). To this end, the current study measured mental health and social factors among 180 Australian couples during the antenatal period of pregnancy. Mothers were also interviewed 6 weeks’ postpartum. Questionnaires were administered measuring couples’ mental health, social support, relationship quality, and sleep patterns. For fathers, we hypothesised AND would be higher among men experiencing low social support (O’Brien et al. 2017), lower relationship quality (Gawlik et al. 2014; Chhabra et al. 2020), and poor quality of sleep (Hall et al. 2017; Wynter et al. 2020). For mothers, we hypothesised that low paternal antenatal social support would impact significantly on maternal mental health antenatally and postnatally, when mothers increasingly rely on paternal social support. We further hypothesised that paternal AND would predict lower maternal relationship quality and higher maternal AND and PND (O’Brien et al. 2017). Although our primary focus was regarding AND and PND, we also explored whether these social factors also predicted anxiety, stress, and psycho-social well-being. Providing insights into the underlying factors contributing to paternal AND is likely to inform not only paternal mental health in the early stages of fatherhood, but also maternal and infant mental health.

## Methods

### Setting

This study was based at the Mater Mothers Hospital (MMH) in Brisbane, Australia. This tertiary healthcare organisation has public and private hospitals where over 10,000 babies are born annually.

### Participants

The Queensland Family Cohort (QFC) is a prospective longitudinal cohort study with families seen during antenatally and postnatally. The full QFC study and its recruitment of participants have been previously described (Borg et al. 2021). For this analysis, a subset of families experiencing a pregnancy at the MMH were eligible for recruitment from 12 to 24 weeks’ gestation between August 2018 and September 2019. Consenting families (single parent units or mother and partners) were asked to fill in questionnaires and provide biological samples at 24, 28, and 36 weeks of gestation and 6 weeks’ postpartum and from partners, at 24 weeks of gestation only. For this analysis, we included families with mothers and whose partners identified as male. Maternal and paternal characteristics including demographic information (age, ethnicity, education, income, health insurance coverage), were self-reported in the enrolment questionnaire between 20 and 22 weeks (Tables 1 and 2). Questionnaire data from both parents were collected at 24 weeks’ gestation and maternal information at 6 weeks’ postpartum included mental health and well-being, social support, relationship quality, and physical pain, described in measures below. The study was approved by the Mater Misericordia Limited Human Research Ethics and Governance Safety Committee (HREC/16/MHS/113).

**Table 1:** Maternal sample demographics and descriptive statistics (*N* = 180)

Age (in years)	Mean	SD	Range				
	31.89	4.99	19–45				
BMI	Mean	SD	Range				
	27.24	5.05	19.48–44.49				
Parity	0	1	2	3			
Frequencies	13	67	19	7			
Planned pregnancy	Yes	No					
Frequencies	136	38					
Private health	Yes	No					
Frequencies	136	38					
Household income	< 50 k	50–100 k	> 100 k				
Frequencies	19	57	65				
Education	Secondary	Certificate	Diploma	Bachelors	Masters	PhD	Trade
	7	26	18	88	28	8	2

**Table 2:** Paternal sample demographics and descriptive statistics (*N* = 180)

Age (in years)	Mean	SD	Range				
	33.57	5.60	19–50				
BMI	Mean	SD	Range				
	27.14	4.30	17.78–47.71				
Education	Secondary	Certificate	Diploma	Bachelors	Masters	PhD	Trade
	19	32	20	61	26	5	13
Household income	< 50 k	50–100 k	> 100 k				
Frequencies	19	57	65				

### Measures

#### Depression, anxiety, and stress scale (DASS-21)

The DASS-21 contains three self-report scales that measure states of depression, anxiety, and stress using 7-item scales with higher scores (rates from 1 = never to 4 = almost always) reflecting greater depression, anxiety, and stress. For depression, scores 5–6 are mild, 7–10 are moderate, and 11 or above are severe. For anxiety, scores 4–5 are mild, 6–7 are moderate, and 8 or above are severe. For stress, scores 8–9 are mild, 10–12 are moderate, and 13 or above are severe. This test has strong internal reliability, with Cronbach’s alpha coefficients being 0.91, 0.84, and 0.90 for depression, anxiety, and stress, respectively (Lovibond and Lovibond 1995). Among fathers surveyed antenatally, Cronbach’s alpha coefficients were 0.84, 0.72, and 0.86 for depression, anxiety, and stress, respectively. Among mothers surveyed antenatally, Cronbach’s alpha coefficients were 0.79, 0.47, and 0.86 for depression, anxiety, and stress, respectively. Among mothers surveyed postnatally, Cronbach’s alpha coefficients were 0.86, 0.76, and 0.88 for depression, anxiety, and stress, respectively.

#### Edinburgh Postnatal Depression Scale (EPDS)

The EPDS is a 10-item scale that measures emotional and psychological distress among pregnant women and mothers. Higher scores reflect greater depression. The scale has strong internal reliability (*α* = 0.87) where scores of 13 or above indicate a high likelihood that mothers are depressed (EPDS; Cox et al. 1987). In the current study, mothers surveyed postnatally had a high Cronbach alpha coefficient for the scale (*α* = 0.86).

#### Assessment of Quality of Life Instrument (AQoL-6D)

The AQoL-6D is composed of six dimensions (1, independent living; 2, relationships; 3, mental health; 4, coping; 5, pain; 6, senses). Higher scores represent worsening psycho-social well-being and physical pain (AQoL-6D; Richardson et al. 2012). Dimensions 1–3 were combined to create a psycho-social well-being dimension (*α* = 0.93), and dimensions 4–6 were combined to create a physical pain dimension (*α* = 0.87; Allen et al. 2013). The Cronbach alpha coefficient for the psycho-social well-being dimension was 0.81, and physical pain was 0.75 for fathers surveyed antenatally. For mothers surveyed antenatally, the Cronbach alpha coefficient for the psycho-social well-being dimension was 0.80 and physical pain was 0.80. Among mothers surveyed postnatally, Cronbach’s alpha coefficient for the psycho-social well-being dimension was 0.83 and physical pain was 0.74.

#### Multidimensional Scale of Perceived Social Support (MSPSS)

This 12-item scale quantifies perceived support across three domains: family, friends, and a significant other using a 7-point scale. Higher scores reflect greater perceived social support. Internal reliability for these domains were high, with Cronbach’s alpha coefficients being 0.87, 0.85, and 0.91 for family, friends, and a significant other, respectively. The Cronbach’s alpha coefficient for the total scale was 0.88 (MSPSS; Zimet et al. 1988). In the current study, Cronbach’s alpha coefficients for the total scale were 0.95, 0.96, and 0.96 for fathers surveyed antenatally, mothers surveyed antenatally, and mothers surveyed postnatally, respectively.

#### Couples Satisfaction Index (CSI-16)

The CSI-16 is a 16-item measure designed to assess relationship satisfaction of intact (married, cohabiting, or dating) couples using a 6-point scale. Internal reliability for this scale is very high (*α* = 0.98), which in the current study during antenatal surveys among fathers was *α* = 0.63 and mothers antenatally was *α* = 0.56 and postnatally was *α* = 0.74. This measure includes items aimed at assessing the presence of problems between individuals and the intensity of such problems using a six-point scale (CSI-16; Funk and Rogge 2007).

### Data analysis

Measures of mental health (DASS-21, EPDS, and psycho-social well-being) were continuous dependent variables in univariate linear regressions wherein measures of social support (MSPSS), relationship quality (CSI-16), sleep patterns, and quality of life (AQoL-6D) were predictor variables. Data for women and men were analysed separately. After identifying univariate predictors between measures of mental health and other dependent variables (retaining only those predictors at *p* < 0.05), multiple regressions were undertaken. All continuous data were mean-centred and transformed into *Z*-scores prior to running the regressions in R (R Core Team 2013).

## Results

A total of 180 women (mean age = 31.89 years, *sd* = 4.99) and 180 men (mean age = 33.57 years, *sd* = 5.6) participated in the study. Participants were well-educated, with 71% having a tertiary education. A total of 57% of couples had private health coverage and 43% did not (Tables 1 and 2). Antenatal depressive symptoms (measured using the DASS-21) were significantly higher among men (mean = 5.38) than women (mean = 3.15), *t*(215.38) = 3.34, *p* < 0.001, *d* = 0.43. While other sex differences were not statistically significant, anxiety symptoms were higher among men (mean = 4.16) than women (mean = 3.51), *t*(205.7) = 1.13, *p* = 0.261, *d* = 0.15, as were stress-related symptoms in men (mean = 8.98) than women (mean = 7.46), *t*(235.17) = 1.65, *p* = 0.100, *d* = 0.21.

### Paternal antenatal mental health

#### Univariate regressions

Paternal depression, anxiety, stress (measured using the DASS-21), and psycho-social well-being were entered as independent variables in univariate regressions. Fathers reporting higher depression, anxiety, stress, and worse psycho-social well-being reported significantly lower social support and higher chronic pain (all *p*s < 0.001). Men with higher depression, anxiety, stress, and worse psycho-social well-being also had lower relationship quality (all *p*s < 0.05). Fathers reporting higher depression, stress, and worsening psycho-social well-being also reported significantly greater sleep impairment (all *p*s < 0.001), while the association between anxiety and sleep impairment were slightly above statistical significance (*p* = 0.053). None of the associations between paternal mental health and maternal mental health were statistically significant (all *p*s ≥ 0.098). The full analyses are in the ESM 1 (Table S1-4).

#### Multiple regressions

Paternal antenatal depression (measured using the DASS-21) was the dependent variable (DV) and relationship quality, sleep impairment, physical pain, and social support were independent variables (IV) in a significant regression, *F*(4,90) = 10.55, *p* < 0.001, *R*^2^ = 0.32. Depression was higher among men reporting greater sleep impairment (*β* = 0.26, *p* < 0.01) and lower social support (*β* =  − 0.32, *p* < 0.01). Associations were not significant between depression and relationship quality (*β* =  − 0.04, *p* = 0.626) and physical pain (*β* = 0.14, *p* = 0.127).

When paternal anxiety was a DV, analyses included physical pain, social support, and relationship quality as IVs, *F*(3,93) = 8.63, *p* < 0.001, *R*^2^ = 0.22. Anxiety was significantly higher among men reporting greater physical pain (*β* = 0.19, *p* = 0.042) and lower social support (*β* =  − 0.30, *p* = 0.003). Relationship quality did not predict anxiety (*β* =  − 0.01, *p* = 0.942).

Analyses including paternal stress as the DV and sleep impairment, physical pain, and social support as IVs revealed a significant regression equation, *F*(4,93) = 8.00, *p* < 0.001, *R*^2^ = 0.26. Stress was higher among men reporting greater sleep impairment (*β* = 0.32, *p* < 0.001). Relationship quality (*β* =  − 0.10, *p* = 0.330), physical pain (*β* = 0.19, *p* = 0.074), and social support (*β* =  − 0.18, *p* = 0.105) were not significant predictors of stress.

Finally, analyses wherein paternal psycho-social well-being was the DV included sleep impairment, physical pain, social support, and relationship quality as IVs, *F*(3,92) = 14.46, *p* < 0.001, *R*^2^ = 0.39. Psycho-social well-being was worse among men with greater sleep impairment (*β* = 0.30, *p* < 0.001) and physical pain (*β* = 0.32, *p* < 0.001), as well as lower social support (*β* =  − 0.31, *p* = 0.001). Relationship quality was not a significant predictor of men’s psycho-social well-being (*β* = 0.01, *p* = 0.880).

### Maternal antenatal mental health

#### Univariate regressions

Depression was significantly higher among mothers reporting lower relationship quality and social support (all *p*s < 0.05). Social support was also significantly lower among mothers reporting worsening psychosocial well-being (*p* = 0.009). Although social support was reportedly lower among mothers with higher anxiety and stress, these associations did not reach statistical significance (all *p*s ≥ 0.086). Lower quality of sleep was higher among mothers reporting higher depression, anxiety, stress, and worse psycho-social well-being (all *p*s < 0.01). Chronic pain was significantly higher among mothers reporting higher depression, anxiety, stress, and worse psycho-social well-being (all *p*s < 0.001). Relationship quality was not significantly associated with anxiety (*p* = 0.209). Mothers reporting higher stress were currently in relationships with men reporting lower relationship satisfaction (*p* = 0.017), while mothers reporting worsening antenatal psycho-social well-being were in relationships with men reporting higher anxiety (*p* = 0.026). None of the other mental health or social variables among fathers significantly influenced maternal antenatal mental health (all *p*s ≥ 0.061). For the full analyses, see the ESM Table S2.

#### Multiple regressions

When women’s depression (measured using the DASS-21) was the DV, the multivariate regression included relationship quality, sleep impairment, physical pain, and social support as IVs, *F*(4,104) = 6.07, *p* < 0.001, *R*^2^ = 0.16. Depression was higher among mothers with greater physical pain (*β* = 0.27, *p* = 0.006). Relationship quality (*β* =  − 0.19, *p* = 0.060), social support (*β* =  − 0.09, *p* = 0.321), and sleep impairment (*β* = 0.10, *p* = 0.314) were not significant predictors of depression.

Analyses wherein women’s anxiety was the DV included sleep impairment and physical pain revealed a significant regression equation as IVs, *F*(2,111) = 11.21, *p* < 0.001, *R*^2^ of 0.17. Anxiety was significantly positively associated with physical pain (*β* = 0.35, *p* < 0.001), while sleep impairment was not a significant predictor of anxiety (*β* = 0.18, *p* = 0.061).

When maternal stress was the DV, analyses included sleep impairment, physical pain, and relationship quality as IVs, *F*(3,106) = 19.78, *p* < 0.001, *R*^2^ = 0.36. Stress was significantly positively associated with physical pain (*β* = 0.41, *p* < 0.001) and sleep impairment (*β* = 0.30, *p* = 0.001). Relationship quality was not a significant predictor of stress (*β* =  − 0.15, *p* = 0.080). While paternal antenatal relationship quality positively predicted maternal anxiety in univariate tests, this association was not statistically significant in multivariate analyses (*β* =  − 0.16, *p* = 0.079).

Analyses including women’s psycho-social well-being as the DV included relationship quality, sleep impairment, parity, physical pain, and social support as IVs, *F*(5,104) = 16.85, *p* < 0.001, *R*^2^ = 0.45. Psycho-social well-being was worse among mothers with greater sleep impairment (*β* = 0.25, *p* = 0.003), physical pain (*β* = 0.37, *p* < 0.001), and lower relationship quality (*β* =  − 0.24, *p* = 0.003). Associations were not statistically significant between psycho-social well-being and self-reported social support (*β* =  − 0.07, *p* = 0.354) and parity (*β* = 0.02, *p* = 0.742). Although paternal antenatal anxiety (measured using the DASS-21) positively predicted worsening maternal psycho-social well-being in univariate analyses, this relationship was not significant in the multivariate analyses (*β* = 0.01, *p* = 0.915).

### Maternal postnatal mental health

#### Univariate regressions

Postnatal depression, stress, anxiety, psycho-social well-being, and depression measured using Edinburgh Postnatal Depression Scale (EPDS) were higher among mothers reporting lower social support (all *p*s < 0.01). Mothers whose partners reported lower social support also reported higher postnatal depression (*p* = 0.055) and worsening psycho-social well-being (*p* = 0.007). Chronic pain was higher among mothers reporting higher postnatal depression, stress, anxiety, depression measured using the EPDS, and worsening psycho-social well-being (all *p*s < 0.05). Finally, postnatal relationship quality was lower among mothers reporting higher postnatal depression, stress, anxiety, depression measured using the EPDS, and worsening psycho-social well-being (all *p*s < 0.05). No other univariate tests were statistically significant (Table S3).

#### Multiple regressions

Analyses wherein women’s depression (measured using the DASS-21) was the DV included relationship quality, physical pain, and social support as IVs, *F*(3,104) = 9.52, *p* < 0.001, *R*^2^ = 0.22. Depression was significantly higher among mothers with higher physical pain (*β* = 0.22, *p* = 0.012) and lower social support (*β* =  − 0.25, *p* = 0.021), but not relationship quality (*β* =  − 0.16, *p* = 0.107).

In univariate tests, the association between paternal antenatal social support and maternal postpartum depression was only marginally higher that the 5% threshold for conventional statistical significance (*p* < 0.055; Table S3). Thus, we then ran an exploratory analysis, entering paternal antenatal social support as an IV into the regression revealed a significant regression equation (*F*(5,70) = 5.22, *p* < 0.001), *R*^2^ = 0.27. Depression was higher among mothers reporting lower social support (*β* =  − 0.28, *p* = 0.018), while paternal antenatal social support (*β* = 0.12, *p* = 0.323), maternal relationship quality (*β* = 0.15, *p* = 0.202), and maternal chronic pain (*β* = 0.18, *p* = 0.092) were not significant predictors of maternal depression. A significant interaction between paternal antenatal depression and maternal postpartum social support (*β* = 0.22, *p* = 0.050) reflects that the negative association between maternal depression and social support was stronger among mothers whose partners reported lower antenatal social support.

Analyses wherein maternal postnatal anxiety was the DV and relationship quality, physical pain, and social support were the IVs revealed a significant regression equation, *F*(3,105) = 5.81, *p* < 0.001, *R*^2^ = 0.14. Anxiety was higher among mothers reporting higher physical pain (*β* = 0.21, *p* = 0.035), while lower social support (*β* =  − 0.22, *p* = 0.057) and relationship quality (*β* =  − 0.11, *p* = 0.326) did not significantly predict anxiety.

Analyses of maternal postnatal stress as the DV and relationship quality, physical pain, and social support as IVs resulted in a significant regression equation, *F*(3,103) = 16.66, *p* < 0.001, *R*^2^ = 0.33. Stress was significantly higher among mothers reporting lower social support (*β* =  − 0.48, *p* < 0.001). Relationship quality (*β* =  − 0.13, *p* = 0.166) and physical pain (*β* = 0.06, *p* = 0.476) were not significant predictors of stress.

Analyses in which women’s postnatal psycho-social well-being was the DV and relationship quality, physical pain, and social support were IVs revealed a significant regression equation, *F*(3,109) = 33.95, *p* < 0.001, *R*^2^ = 0.48. Psycho-social well-being was significantly worse among mothers with higher physical pain (*β* = 0.36, *p* < 0.001) and lower social support (*β* =  − 0.44, *p* < 0.001). However, relationship quality was not a significant predictor of maternal psycho-social well-being (*β* =  − 0.13, *p* = 0.104).

A regression including paternal antenatal depression measured using the DASS-21 and social support revealed a significant equation, *F*(2,85) = 7.72, *p* < 0.001; *R*^2^ = 0.15. Postnatal psycho-social well-being was worse among mothers whose partners reported higher antenatal depression (*β* = 0.33, *p* = 0.009). Paternal antenatal social support did not predict postnatal maternal psycho-social well-being (*β* =  − 0.09, *p* = 0.467).

Analyses of depression measured using the EDPS as the DV including relationship quality, physical pain, and social support as IVs resulted in a significant regression equation, *F*(3,108) = 13.06, *p* < 0.001, *R*^2^ = 0.25. Depression was significantly higher among mothers reporting greater physical pain (*β* = 0.17, *p* = 0.046) and lower social support (*β* =  − 0.39, *p* < 0.001). Relationship quality was not a significant predictor of maternal depression (*β* =  − 0.09, *p* = 0.368).

## Discussion

Our study contributes new insights into the predictors of impaired mental health among fathers in the antenatal stages and the influences they have on social and mental health among mothers antenatally and postnatally. Antenatally, paternal depression, anxiety, and stress were higher and psycho-social well-being was worse among men reporting lower social support, poor sleep patterns, and in some cases, lower relationship quality and greater physical pain. Like fathers, maternal antenatal mental health was lower among mothers reporting poorer sleep patterns and higher physical pain. Antenatally, low social support, physical pain, and to some extent low relationship quality predicted lower maternal mental health and well-being. Our exploratory analyses also revealed that mothers in relationships with fathers reporting low antenatal social support from friends, family, and significant others, in turn, reported lower postnatal social support and higher depression. We suggest a whole family focus on mental health and social support during and after the transition to parenthood is necessary.

Social network theory suggests mental health and emotional well-being are improved through access to support from immediate and extended family members and friends (Valente and Pitts 2017). Previous studies reported social support was a robust predictor of maternal depression at both the antenatal (Recto and Champion 2020) and postnatal phases (Gao et al. 2009; Rao et al. 2020) and that interventions on antenatal social support can lower depression by 3% (Spry et al. 2021). In our sample of Australian mothers, multivariate analyses revealed no evidence that maternal antenatal mental health was lower among mothers with lower self-reported social support. However, among fathers, low social support was a significant predictor of paternal antenatal depression, anxiety, and psycho-social well-being. Although scarce, there is some evidence that Chinese fathers reporting low social support had higher postnatal depression (Gao et al. 2009; Mao et al. 2011) and fathers from the USA named fewer family members, smaller social support networks, and lower support from family members than mothers (Oliveri and Reiss 1987). Thus, social groups among fathers may be incompatible with the demands of fatherhood and family life (Glasser, and Lerner-Geva 2019), leaving men potentially vulnerable to a lack of social support during the critical early stages of family life.

While maternal antenatal mental health in our sample was comparatively less impacted by social support than among fathers, postnatal mental health symptoms were higher among mothers reporting lower social support. Previous research has shown that mothers who maintain their social group memberships prior to pregnancy and motherhood have lower postnatal depression (Seymour-Smith et al. 2017). Our data were collected in the early weeks postpartum, when mothers may not be able to access their broader support networks and subsequently turn to fathers for additional support. However, men may require support from their partners as they also become detached from their extended social networks during the transition to fatherhood (Fletcher et al. 2014). The significant interaction we report between maternal postnatal social support and paternal antenatal social support in predicting women’s postpartum depression suggests the need for social support screening and intervention among fathers during the early antenatal period to support their partners (O’Brien et al. 2017). However, it is important to note that these analyses are exploratory and require further replication.

As in past research, we report antenatal depression and stress were higher among mothers and fathers with poor sleep quality antenatally (Giallo et al. 2013; Smart and Hiscock 2007; Krueger and Friedman 2009), which is concerning as sleep quality may decline further postpartum. Indeed, sleep impairment is common among fathers from birth, continues up to 1 year of parenthood (Wynter et al. 2020), and is associated with higher depression and anger towards their children (Cook et al. 2017). Poor sleep quality is also associated with physical pain that further affects parental mental health (Krueger and Friedman 2009), and the impact of physical pain on worsening maternal well-being during and after the transition the motherhood is well established (Ray-Griffith et al. 2018). Our study is consistent with these findings, such that physical pain positively predicted maternal antenatal depression, anxiety, stress, and worse psycho-social well-being in both univariate and multivariate analyses. However, there is comparatively less evidence that physical pain might influence paternal mental health (Bakermans-Kranenburg et al. 2019), and we found that paternal physical pain was positively associated with antenatal anxiety and worse psycho-social well-being, suggesting the need for further research into men’s physical health during the transition to fatherhood.

There are some important limitations to our study. While our findings suggest paternal social support may impact maternal mental health during the early months of motherhood, most of the couples in our study were well educated and of middle-class socioeconomic status from a large metropolitan city. Expanding data collection to include parents from a broader cross section of socioeconomic backgrounds, family structures, and same-sex couples would inform the generalisability of our findings. Another important limitation in our study was the lack of data from fathers at the postnatal phase, and further replication employing samples collected from both parents at the antenatal and postnatal stages would be valuable (Kamalifard et al. 2014; Clifton et al. 2022).

Although screening for mental health during the antenatal and postnatal periods is common for mothers, it is rarely undertaken among fathers at either the perinatal or antenatal stages. A confluence of social and economic factors could influence men’s transitions to parenthood, including conflicts between professional work and investment in the family, and social expectations surrounding hegemonic and traditional forms of masculinity with domestic divisions of labour and co-parenting (Gatrell et al. 2022). Maternal preferences for masculine partners may vary when considering long-term and investing co-parents than a less parentally investing partner (Clarkson et al. 2020; Dixson et al. 2018, Stower et al. 2020) particularly among mothers with young children (Buchler et al. 2017; Dixson et al. 2019). Our findings suggest that men’s depression, anxiety, and stress during the antenatal stage of their partner’s pregnancy are associated with lower self-reported social support. We recommend mental health and social support screening during the antenatal phase among fathers and mothers, as well as social support intervention for fathers when needed. Future clinical interventions could also consider men’s social support and willingness to access healthcare services during the transition to fatherhood (Bateson et al. 2017). For the present, our findings suggest that accounting for father’s antenatal mental health and social support is necessary to achieve a whole family approach to well-being during the transition to parenthood.

## Supplementary Information

ESM 1 (XLSX 24.5 KB)

## Data Availability

Data are available on request from Mater Misericordia Limited Human Research Ethics and Governance Safety Committee.
